# SUMOylated non-canonical polycomb PRC1.6 complex as a prerequisite for recruitment of transcription factor RBPJ

**DOI:** 10.1186/s13072-021-00412-9

**Published:** 2021-07-31

**Authors:** Małgorzata Sotomska, Robert Liefke, Francesca Ferrante, Heiko Schwederski, Franz Oswald, Tilman Borggrefe

**Affiliations:** 1 Institute of Biochemistry, Justus-Liebig University of Giessen, Friedrichstrasse 24, 35392 Giessen, Germany; 2 Institute of Molecular Biology and Tumor Research (IMT), Philipps University of Marburg, Hans-Meerwein Strasse 2, 35043 Marburg, Germany; 3 Department of Hematology, Oncology and Immunology, University Hospital Marburg and Philipps University of Marburg, Baldingerstrasse, 35043 Marburg, Germany; 4 Center for Internal Medicine, Department of Internal Medicine 1, University Medical Center Ulm, Albert-Einstein-Allee 23, 89081 Ulm, Germany

**Keywords:** Notch signaling, Polycomb repressive complex, RBPJ, Sumoylation, Epigenetics

## Abstract

**Background:**

Notch signaling controls cell fate decisions in many contexts during development and adult stem cell homeostasis and, when dysregulated, leads to carcinogenesis. The central transcription factor RBPJ assembles the Notch coactivator complex in the presence of Notch signaling, and represses Notch target gene expression in its absence.

**Results:**

We identified L3MBTL2 and additional members of the non-canonical polycomb repressive PRC1.6 complex in DNA-bound RBPJ associated complexes and demonstrate that L3MBTL2 directly interacts with RBPJ. Depletion of RBPJ does not affect occupancy of PRC1.6 components at Notch target genes. Conversely, absence of L3MBTL2 reduces RBPJ occupancy at enhancers of Notch target genes. Since L3MBTL2 and additional members of the PRC1.6 are known to be SUMOylated, we investigated whether RBPJ uses SUMO-moieties as contact points. Indeed, we found that RBPJ binds to SUMO2/3 and that this interaction depends on a defined SUMO-interaction motif. Furthermore, we show that pharmacological inhibition of SUMOylation reduces RBPJ occupancy at Notch target genes.

**Conclusions:**

We propose that the PRC1.6 complex and its conjugated SUMO-modifications provide a favorable environment for binding of RBPJ to Notch target genes.

**Supplementary Information:**

The online version contains supplementary material available at 10.1186/s13072-021-00412-9.

## Background

Notch signal transduction is an evolutionary conserved pathway that regulates stem cell maintenance and differentiation decisions throughout development. Dysregulation of either NOTCH receptors or their modifiers are linked to carcinogenesis [1–3]. At the molecular level, ligand binding leads to the proteolytic processing of the Notch receptor. Its intracellular domain migrates then into the nucleus, associates with the transcription factor RBPJ and activates transcription of target genes. In the absence of a Notch signal, RBPJ assembles an HDAC-containing corepressor complex and represses transcription. The Notch transcriptional signature is quite diverse in different tissues and cell types, even in related cell types, such as B- and T-cells. Transcription factor in concert with chromatin modifiers establish a chromatin environment that predetermines specificity of the Notch target gene expression [3–5]. In regard to posttranslational modifications (PTMs), not only histone tails, but also the Notch coactivator complex are known to be phosphorylated, methylated, acetylated, SUMOylated and ubiquitinylated [6–8]. These modifications control not only the stability of Notch, but also determine amplitude and duration of the Notch response [9] and can be incorporated upon cellular stress signals [10].

The central player controlling the expression of Notch target genes is the transcription factor RBPJ, also known as CBF1 (C promoter binding factor) or CSL (*Homo sapiens* CBF1, *Drosophila melanogaster* Suppressor of Hairless, and *Caenorhabditis elegans* Lag-1). RBPJ binds to the sequence 5′-CGTGGGAA-3′ [11] and its genome-wide distribution has been studied in several tissues [12]. In the absence of a Notch signal, RBPJ assembles a corepressor complex containing NCoR/HDACs [13] and histone demethylases, such as KDM1A/LSD1 [14].

Polycomb group proteins assemble in two major repressive multi-subunit complexes known as PRC1 (Polycomb repressive complex 1) and PRC2. PRC1 and PRC2 differ in their enzymatic activities and function. PRC1 contains the E3 ligase RING1/2 and PRC2 the repressing histone methyltransferase EZH2. The PRC1-components relevant for this study (L3MBTL2, MGA and E2F6) are subunits of the PRC1.6 complex described by Trojer [15]. PRC1.6 belongs to the group of non-canonical PRC1, which are known to be recruited also in an H3K27me3-independent manner [16, 17]. The genome-wide distribution of PRC1.6 has been studied using CRISPR-Cas9 depletion of the individual components L3MBTL2, E2F6, MGA and PCGF6 combined with ChIP-Seq analyses of the same proteins, revealing that binding of PRC1.6 components overlap genome-wide and that L3MBTL2 and E2F6 contribute to chromatin binding of the PRC1.6 complex [18]. In addition, SUMOylation of L3MBTL2 is required for transcriptional repression of endogenous target genes [19].

SUMO (small ubiquitin-like modifier) is a 11 kDa protein that modifies its target proteins through covalent binding to lysine residues [20]. The family of SUMO proteins consists of three evolutionary conserved, functional isoforms (SUMO1, SUMO2 and SUMO3) of which the last two are 97% identical and are, therefore, often referred to as SUMO2/3 [21]. SUMO is attached to proteins using E1 (Aos1/Uba2), E2 (Ubc9), and several E3 ligase enzymes. There are also proteins that bind SUMO non-covalently by specific SUMO interacting motifs (SIMs), implicating that SUMO and SIM-containing proteins can form multiprotein networks [22]. In consequence, SUMO modifications are not only regulating the functions of individual proteins, but also regulate protein complex assembly and recruitment [23–25]. Importantly, Cossec and colleagues revealed that chromatin-bound SUMO and the PRC1.6 complex, in particular L3MBTL2, are found at the overlapping genomic sites. It is reported that multiple SUMO-moieties act as a “glue” for both transcription factors and chromatin regulators, thereby stabilizing key determinants of somatic pluripotent states [26].

Here, we show that components of the PRC1.6 complex together with multiple SUMO-modifications physically interact with the transcription factor RBPJ, thereby supporting its binding to enhancers of Notch target genes. Specifically, we found that L3MBTL2 and E2F6 are required for efficient RBPJ binding. Furthermore, we reveal that RBPJ utilizes its SUMO interaction motif as additional docking site and provide evidence that high local SUMO levels and occupancy of the PRC1.6 complex are favorable for RBPJ binding.

## Results

### DNA-bound RBPJ is associated with several PRC1.6 components

**Fig. 1:**
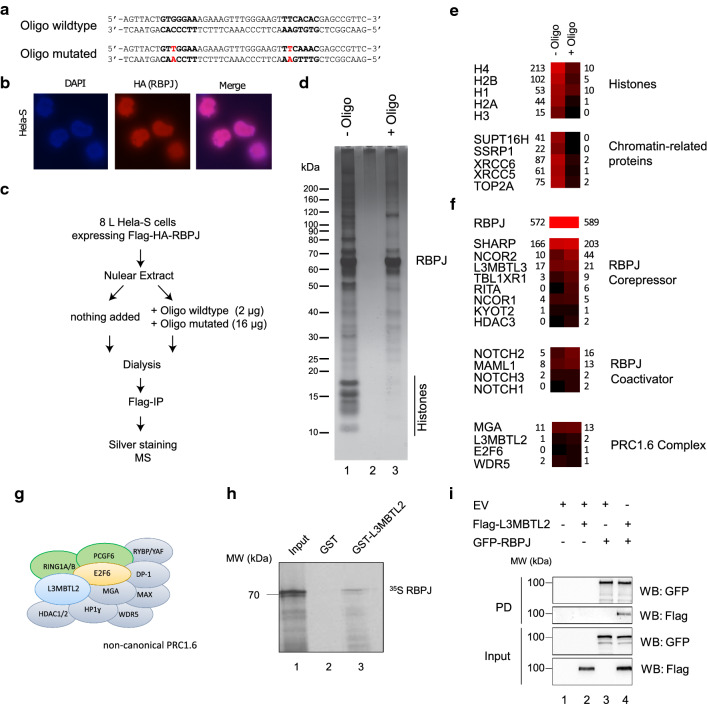
Oligonucleotide-assisted complex purification of RBPJ and validation of a direct RBPJ-L3MBTL2 interaction. **a** Oligonucleotides used to stabilize RBPJ complexes during purification. The sequence of the double-stranded oligo is based on the *Hes1* promoter as described in [27]. **b** Immunofluorescence of Flag-HA-tagged RBPJ expressed in HeLa-S cells. **c** Experimental outline of oligonucleotide-assisted complex purification of Flag-HA-RBPJ from HeLa-S cells. **d** Silver staining of purified RBPJ complexes, obtained in the presence and absence of the oligonucleotides. **e** Example proteins, that are strongly reduced in the RBPJ complex purified in presence of the oligonucleotides. **f** Proteins associated with the RBPJ coactivator or corepressor complexes. Components of the PRC1.6 are putative novel RBPJ-associated proteins. The numbers in **e** and **f** indicate the total peptide numbers identified by mass-spectrometry (see also Additional file 1: Table S1). **g** Schematic representation of PRC1.6 subunits [16]. **h** GST-pulldown assays were performed with GST-L3MBTL2 or GST only and [^35^S] methionine-labelled RBPJ. Bound proteins were separated in SDS-PAGE and visualized by autoradiography. **i** Co-Immunoprecipitation experiments were performed using Flag-L3MBTL2 and GFP-RBPJ overexpressed in HEK293T cells. GFP-RBPJ and Flag-L3MBTL2 were expressed in 293T cells. Lysates were subjected for GFP immunoprecipitation, followed by Western blotting. Control cells were transfected with pcDNA GFP plasmid

To biochemically isolate RBPJ-containing complexes, we took advantage of a DNA double-stranded oligonucleotide containing two RBPJ binding sites 5′-GTGGGAA-3’ (Fig. 1a), which allows the formation of DNA-bound dimeric RBPJ complexes [27]. We speculated that DNA-binding of RBPJ may stabilize the complex and in addition prevent unspecific binding of proteins to the charged DNA-binding region of RBPJ, similar to the previously described trapping method [28].

Nuclear extracts from HeLa cells expressing FLAG-HA-tagged RBPJ (Fig. 1b) were generated by salt extraction. Subsequently, we added the double-stranded oligonucleotide prior to dialysis. To prevent binding of unspecific DNA binding proteins to the RBPJ-bound DNA, we additionally added a mutated oligonucleotide (Fig. 1a) in excess. In a control experiment, we did not add any DNA to the samples. After the dialysis step, we isolated the complexes using Flag-beads (Fig. 1c). When analysing the bound proteins by silver staining, we found that adding the oligonucleotide (wild type and mutated for the RBPJ binding motif) strongly reduced the level of unspecific proteins (Fig. 1d). In particular, the amount of histone proteins was strongly reduced in the DNA-treated sample as compared with the untreated sample (Fig. 1d). Subsequently, the obtained protein samples were subjected to mass-spectrometric analysis. The MS data confirmed the strong reduction of histone proteins and chromatin-associated proteins in the DNA-bound RBPJ sample (Fig. 1e, Additional file 1: Table S1). Of note, RBPJ itself, and known RBPJ interactors either were not affected by adding the DNA or we obtained even higher peptide numbers (Fig. 1f). Thus, by adding the RBPJ-binding oligonucleotide during the RBPJ complex purification procedure, we substantially improved the signal to noise ratio. Closer inspection of the data showed that we could identify many known RBPJ corepressor and coactivator complex components in the sample, such as NOTCH2, MAML1, SHARP [29], L3MBTL3 [14], RITA [30, 31] and NCoR components [13]. As putative novel components, we detected MGA, L3MBTL2, E2F6 and WDR5 (Fig. 1f), subunits of the PRC1.6 complex (Fig. 1g).

L3MBTL3/MBT1 was described to interact directly with RBPJ [14] in a neuronal context. To investigate whether L3MBTL2 and RBPJ also interact directly, we performed GST pulldown experiments. Bacterially expressed GST-L3MBTL2 or GST-only as control were used as baits and incubated with radioactively labelled RBPJ. RBPJ interacted with GST-L3MBTL2, but not with GST-only (Fig. 1h). To further validate the interaction of L3MBTL2 with RBPJ, we performed co-immunoprecipitation assays. We observed that L3MBTL2 co-immunoprecipitated with GFP-RBPJ (Fig. 1i). Endogenous L3MBTL2 was also detected after GFP-RBPJ immunoprecipitation in HEK293T cells (Additional file 2: Fig. S1a). Next, we mapped the L3MBTL2 domain that binds to RBPJ using four fragments of L3MBTL2. Two of them contained MBT domains, L3MBTL2 ∆N and L3MBTL2 ∆C, and two of them contained only the N- or the C-terminal part, L3MBTL2-N and L3MBTL2-C. The GST pull-down experiments revealed that the four central MBT domains of L3MBTL2 are required for the interaction with RBPJ (Additional file 2: Fig. S1b). Furthermore, we can show that the C-terminal end of L3MBTL2 is sufficient for the interaction with RBPJ in cells (Additional file 2: Fig. S1c). We also mapped the RBPJ domain that interacts with L3MBTL2 using three overlapping fragments of RBPJ: RBPJ N/B, which contains both NTD (N-Terminal Domain) and BTD (Beta Trefoil Domain) domains, RBPJ B/C containing BTD and CTD (C-Terminal Domain), and RBPJ B, which is almost completely restricted to the BTD. Only RBPJ N/B interacted with L3MBTL2 (Additional file 2: Fig. S1d), suggesting that L3MBTL2 interacts exclusively with N-terminal part of RBPJ. To investigate if the NTD fragment of RBPJ is sufficient for the interaction with L3MBTL2, we used it in GST pull-down experiment with GST-L3MBTL2. Indeed GST-L3MBTL2 is able to interact with RBPJ NTD (Additional file 2: Fig. S1e). This finding was unexpected, since most of the interactions of RBPJ with other corepressors, such as KyoT2 or RITA, are within BTD, or such as SHARP, with both BTD, and CTD of RBPJ [29, 31–34]. Together, RBPJ and L3MBTL2 strongly interact confirming the unbiased initial mass spectrometric results.

### Loss of L3MBTL2 or E2F6 functionally affects Notch target gene expression

**Fig. 2:**
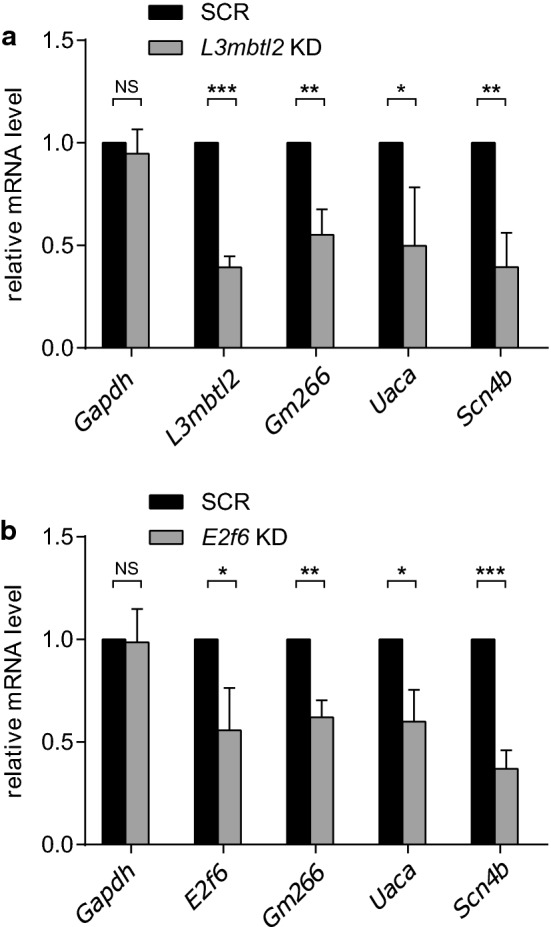
*L3mbtl2* and *E2f6* silencing functionally affects Notch target genes in mouse leukemia pre-T cells. **a** Beko cells were lentivirally infected with indicated shRNAs targeting *L3mbtl2* or **b** *E2f6* gene. 48 h after the last infection, cells were selected with puromycin. Indicated mRNA levels were measured by quantitative real-time PCR. Data was normalised to *Hprt, Gapdh* served as a control. (*p < 0.05, **p < 0.01, ***P < 0.001, [NS] not significant, unpaired Student's *t* test)

To functionally investigate the role of the PRC 1.6 complex at Notch target genes, we performed knockdown experiments of two of its subunits: L3MBTL2 and E2F6. We infected with shRNAs (short hairpin RNA) the previously described mouse Beko T-ALL (T-cell acute lymphoblastic leukemia) cell line, where L3MBTL2 interacts with RBPJ at endogenous level (Additional file 2: Fig. S1f) and that is constitutively active for Notch signal transduction and Notch target gene expression [8, 9, 35]. Knockdown of either L3MBTL2 or E2F6 resulted in downregulation of several Notch target genes: *Uaca*, *Gm266*, or *Scn4b* (Fig. 2a, b). This result was somewhat unexpected, since most of the reports describe the PRC1.6 to support repression rather than activation. To further understand the significance of this finding, we wanted to investigate the occupancy of PRC1.6 at Notch target genes using chromatin-IP (ChIP).

### Co-occupancy of L3MBTL2, E2F6 and RBPJ at Notch target genes

**Fig. 3:**
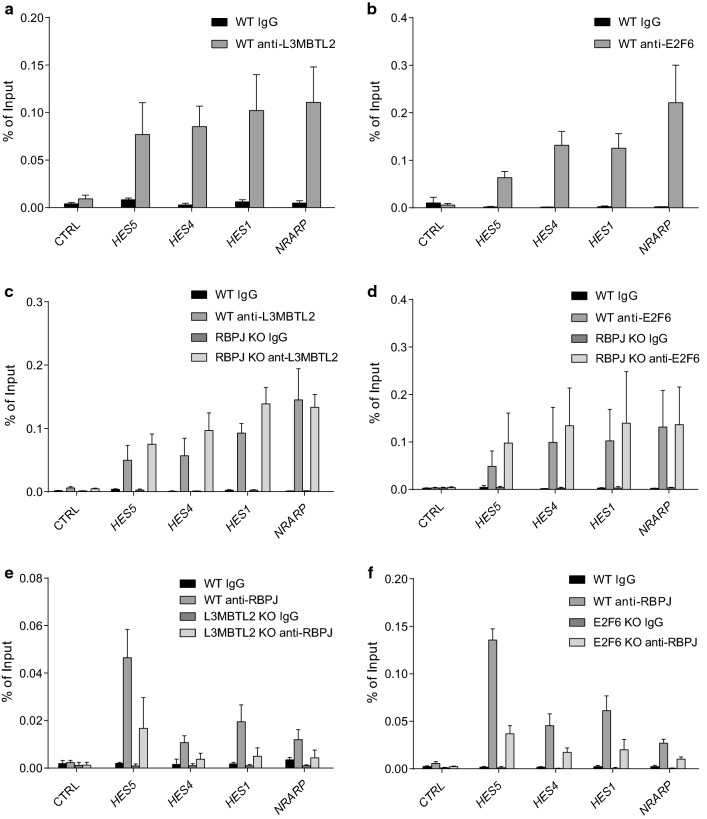
Changes in RBPJ binding depend on the presence of L3MBTL2 and E2F6. **a** ChIP-qPCR experiments showing binding of L3MBTL2 to regulatory elements of Notch target genes in HEK293 cells. *CDC7-2Kb* served as a negative control (CTRL). **b** ChIP-qPCR experiments showing binding of E2F6 to regulatory elements of Notch target genes in HEK293 cells. *Gene Desert* served as a negative control (CTRL). The mean of at least three technical replicates ± SD. **c** ChIP-qPCR analysis of L3MBTL2 binding at regulatory elements of Notch target genes in CRISPR/Cas9 mediated RBPJ depleted cells. **d** ChIP-qPCR analysis of E2F6 binding in CRISPR/Cas9 mediated RBPJ depleted cells in comparison with control cells*. CDC7-2 Kb* served as a negative control (CTRL). **e** ChIP-qPCR analysis of RBPJ binding at regulatory elements of Notch target genes in L3MBTL2 KO and **f** E2F6 KO cells in comparison with the control cells. *Gene Desert* served as a negative control (CTRL). The mean of at least three independent biological replicates ± SD

The genome-wide occupancy of the PRC 1.6 was previously investigated in wild type HEK293 cells and in HEK293 cells, where L3MBTL2, E2F6, MGA or PCGF6 were depleted by CRISPR/Cas9 [18]. Since CRISPR/Cas9 gene editing is so far not possible in Beko T-ALL cells, we switched to the available HEK293 to investigate further the interplay between PRC1.6 components and RBPJ. Importantly, L3MBTL2 (Fig. 3a) as well as E2F6 (Fig. 3b) display strong occupancy at enhancer elements of the well-known Notch target genes *HES1*, *HES4*, *HES5* and *NRARP*. These enhancers are also bound by RBPJ (Additional file 2: Fig. S2a) suggesting co-occupancy of RBPJ and the PRC1.6 complex. Next, we wanted to investigate further the recruitment mechanism, taking advantage of L3MBTL2- and E2F6-depleted HEK293 cells (Additional file 2: Fig. S2b and S2c) [18]. We also generated RBPJ-depleted HEK293 cells using CRISPR/Cas9 with two independent RNA guides (Additional file 2: Fig. S2d). RBPJ depletion was confirmed both on the protein level (Additional file 2: Fig. S2e) and on the mRNA level (Additional file 2: Fig. S2f).

### Binding of RBPJ is impaired in absence of L3MBTL2 or E2F6

First, we investigated whether the occupancy of PRC1.6 components at Notch target genes depends on RBPJ. For this purpose, we used RBPJ-depleted HEK293 cells and performed ChIP with antibodies specific for RBPJ and the PRC1.6 components L3MBTL2 and E2F6. As expected, in RBPJ-depleted cells RBPJ was not detected at enhancers of Notch target genes *HES5*, *HES4*, *HES1* and *NRARP* (Additional file 2: Fig. S2a), showing the specificity of the anti-RBPJ antibody. Next, we investigated PRC1.6 occupancy using anti-L3MBTL2 and anti-E2F6 antibodies. Binding of L3MBTL2 and E2F6 to regulatory elements of the Notch target genes *HES5*, *HES4*, *HES1* and *NRARP* was similar in wild type HEK and in RBPJ-depleted HEK cells (Fig. 3c, d). However, to our surprise, we observed a marked reduction of RBPJ occupancy in L3MBTL2-depleted and in E2F6-depleted cells (Fig. 3e, f). The data suggest that L3MBTL2 and E2F6 support the binding of transcription factor RBPJ to Notch target gene regulatory elements.

### SUMO modifications are present at regulatory elements of Notch target genes

**Fig. 4:**
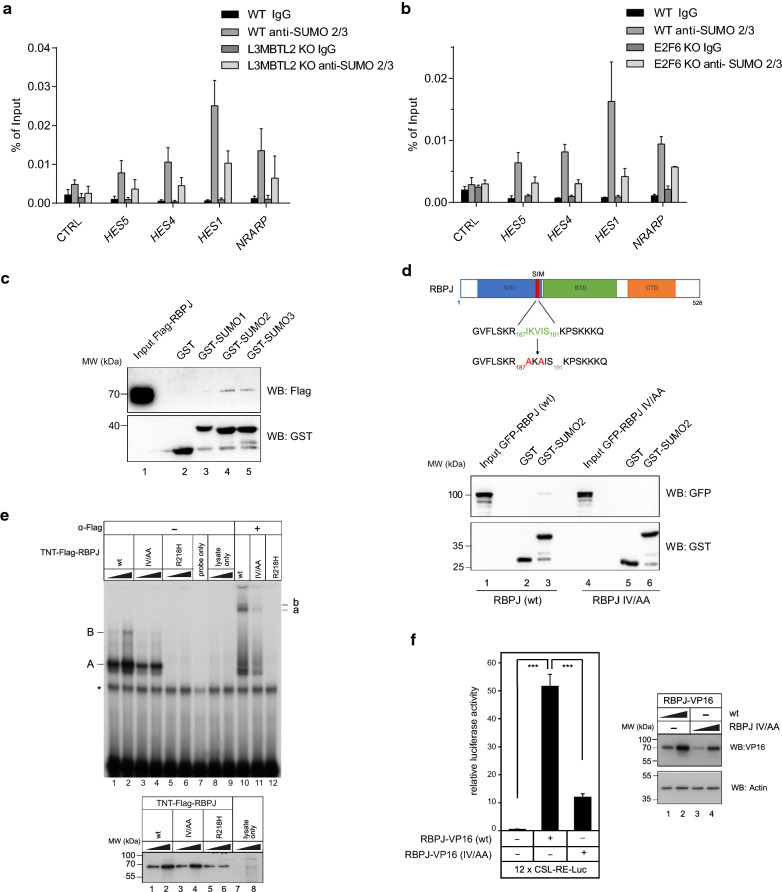
SUMO moieties are found at Notch target genes and are bound non-covalently by RBPJ. **a** ChIP qPCR analysis of SUMO2/3 enrichment at regulatory elements of Notch target genes in HEK293 L3MBTL2 KO or **b** E2F6 KO cells. *Gene Desert* served as a negative control (CTRL). The mean of at least three independent biological replicates ± SD. **c** GST-SUMO1, GST-SUMO2 and GST-SUMO3 fusion proteins were expressed in bacteria and purified. HEK293T cells were transiently transfected with Flag-RBPJ and whole cell extract was incubated with GST fusion proteins immobilized on sepharose beads. Flag-RBPJ binds non-covalently to GST-SUMO2 and GST-SUMO3 but not to GST-only. (**d**, upper) Schematic representation of the wild type and the mutated SIM of RBPJ. (**d**, lower) GST-SUMO2 fusion protein was expressed in bacteria and purified. HEK293T cells were transiently transfected with GFP-RBPJ wild type or GFP-RBPJ IV/AA mutant and whole cell extracts were incubated with GST fusion protein immobilized on sepharose beads. (**e**, upper) Electrophoretic Mobility Shift Assay (EMSA) analysis of RBPJ wt and RBPJ IV/AA mutant binding to DNA. Oligomeric duplex DNA probe with RBPJ binding sites (bold): 5'-CCT GGA ACT ATT **TTC CCA C**GG TGC CCT TCC GCC CAT TTT CCC ACG AGT CG -3'. DNA–protein complexes are indicated as A and B. Supershifted complexes after addition of Flag antibodies are indicated by **a** and **b**. The asterisk indicates a nonspecific background band. (**e**, lower) Western blot showing the in vitro translated Flag-RBPJ proteins used in the EMSA. (**f**, left) Transactivation capacities of RBPJ-VP16 fusion proteins. Hela cells were cotransfected with either RBPJ-VP16 wt or RBPJ-VP16 IV/AA mutant together with 12 × CSL-RE-Luc reporter construct containing 12 RBPJ DNA binding sites upstream of the luciferase gene. The mean of at least four independent biological replicates ± SD is shown (***p < 0.0001, unpaired students T-test). (**f**, right) Western blot show slightly reduced expression of the RBPJ-VP16 (IV/AA) mutant. HeLa cells were transiently transfected with RBPJ-VP16 (wt) or RBPJ-VP16 (IV/AA) mutant. 24 h after transfection cells, where lysed and expression of the VP16 were analysed by western blotting. Actin expression served as a loading control

L3MBTL2 is specifically SUMOylated at lysine residues K675 and K700 close to the C-terminus, and the amount of SUMOylated L3MBTL2 is relatively high at steady state [19]. In addition, other subunits of PRC1.6 including E2F6, PCGF6 and MGA are also modified by SUMO [36, 37]. Moreover, SUMO2/3 primarily modifies highly interconnected repressive chromatin complexes [26]. Therefore, we hypothesized that SUMO is present at Notch target genes. Indeed, in ChIP experiments we found strong SUMO 2/3 enrichment at regulatory elements of Notch target genes (Additional file 2: Fig. S3a). Interestingly, L3MBTL2 is modified by SUMO 2/3 but not by SUMO1 [19]. Importantly, occupancy of SUMO2/3 at the Notch target genes was greatly reduced in cells that are devoid of L3MBTL2 or E2F6 (Fig. 4a, b). Therefore, we reasoned that RBPJ might have an intrinsic capability to recognize SUMO and/or L3MBTL2/E2F6 resulting in RBPJ binding at Notch target genes.

### Identification of RBPJ SUMO interacting motif

To test whether RBPJ can interact with SUMO moieties, we employed GST pulldown experiments using GST-SUMO1, GST-SUMO2, and GST-SUMO3 fusion proteins. RBPJ bound SUMO2 and SUMO3 and to a minor extent also to SUMO1 (Fig. 4c). Furthermore, we can demonstrate that an RBPJ mutant lacking NTD (the region, where L3MBTL2 binds) is not able to bind GST SUMO2 (Additional file 2: Fig. S3b). Subsequently, we searched for a SIM (SUMO Interaction Motif) in the RBPJ sequence using a SUMO/SIM prediction tool [38]. This search identified a putative SIM consensus motif at positions 187–191 in the N-terminal domain (NTD) of RBPJ (Fig. 4d, upper panel). We generated a double-point-mutant form of RBPJ in which I187 and V189 were replaced by alanine residues (further referred as RBPJ IV/AA mutant), to disrupt the hydrophobic core of the SIM consensus (Fig. 4d, upper panel). Next, we tested this mutant in GST pulldown using GST-SUMO2 fusion protein, and we found that RBPJ IV/AA mutant has significantly less binding to SUMO2 in comparison to the wild type RBPJ (Fig. 4d, lower panel). To determine if RBPJ IV/AA mutant retain the ability to functionally bind to DNA, we performed Electro Mobility Shift Assay (EMSA) and luciferase assays. Protein levels of either wild-type RBPJ or IV/AA used for EMSA were similar, whereas RBPJ R218H mutant (DNA-binding deficient) was more unstable (Fig. 4e, lower panel). The DNA binding ability of RBPJ IV/AA mutant was slightly reduced compared to wild-type-RBPJ (Fig. 4e, upper panel, lane 3 and 4). This was also reflected in luciferase assays, where RBPJ IV/AA mutant fused to strong activation domain VP16 was still able to transactivate, though to a lesser extent (Fig. 4f). In addition, the RBPJ IV/AA mutant protein localises in the nucleus as the wild type protein and is still able to mediate NICD-dependent transactivation in reporter gene assays (Additional file 2: Fig. S3c and S3d). However, overall expression after transient transfection appeared to be slightly reduced (Fig. 4f, Additional file 2: Fig. S3c and S3d). Together, RBPJ is able to bind SUMO2 via a SIM located at the end of the N-terminal domain.

### SUMOylation levels play a role in RBPJ recruitment

**Fig. 5:**
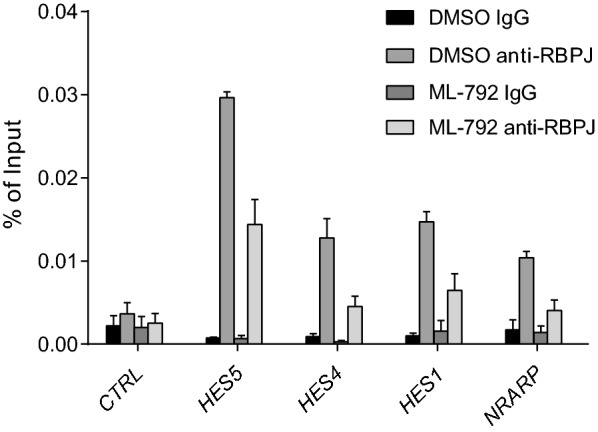
RBPJ recruitment is impaired upon SUMO inhibition. ChIP qPCR analysis of endogenous RBPJ enrichment at regulatory elements of Notch target genes in HEK293 upon 24 h treatment with 10 µM ML-792 or the vehicle. *Gene Desert* served as a negative control (CTRL). The mean of at least three independent biological replicates ± SD

To further investigate the role of SUMOylation-dependent RBPJ recruitment we used the SUMOylation inhibitor ML-792, that specifically blocks the E1 enzyme of the SUMO pathway (Additional file 2: Fig. S4a). We performed ChIP q-PCR experiments with HEK293 cells treated with ML-792 or vehicle as a control. Compared to vehicle treated cells, RBPJ occupancy at Notch target genes was reduced in cells treated with SUMO inhibitor (Fig. 5). In line with this observation, the interaction between Flag-L3MBTL2 and GFP-RBPJ was reduced in the presence of ML-792 (Additional file 2: Fig. S4b). Of note, the interaction of RBPJ with Notch was not affected by ML-792 (Additional file 2: Fig. S4c). Moreover, we used heat shock to increase the level of SUMOylation. Heat shock treated cells show stronger interaction between RBPJ and L3MBTL2 in comparison to untreated cells (Additional file 2: Fig. S4d). In conclusion, SUMOylation stabilizes the interaction of RBPJ with PRC1.6 resulting in stronger binding of RBPJ to Notch target genes.

## Discussion

**Fig. 6:**
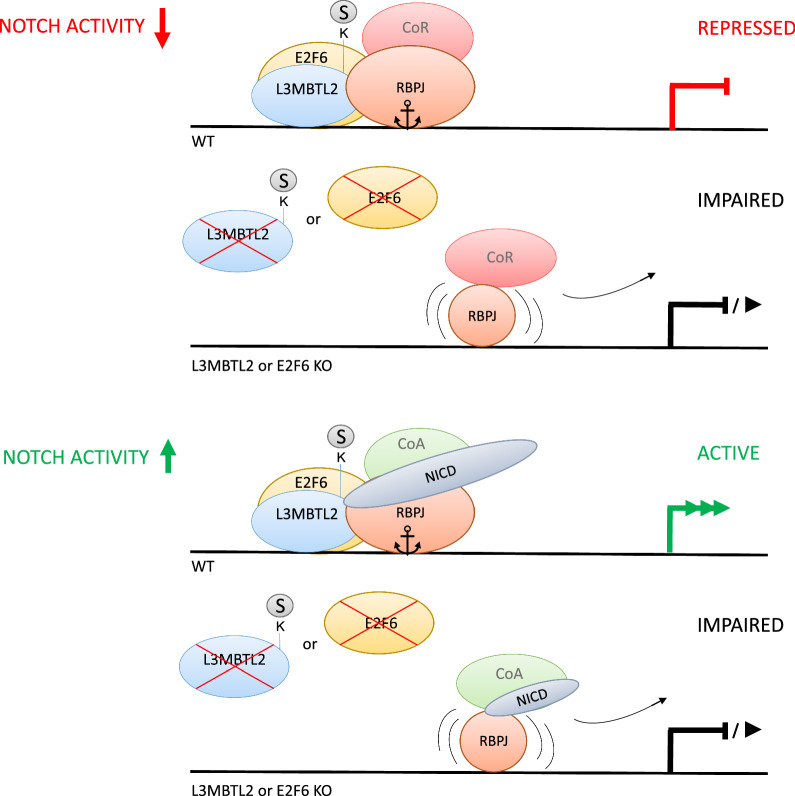
Model for PRC1.6 function in cells with low or high Notch activity. L3MBTL2 and E2F6 binding facilitates establishing preferential environment for transcription factor RBPJ occupancy. In presence of a Notch signal and PRC1.6 complex, RBPJ is properly anchored and can efficiently transcribe (first lane). In the absence of either L3MBTL2 or E2F6 RBPJ chromatin binding is impaired and gene expression is downregulated (second lane). In cells with low Notch signaling activity, RBPJ binds to Notch target genes and represses transcription (third lane). In L3MBTL2 or E2F6-depleted cells, RBPJ occupancy is decreased (fourth lane)

In this study, we identified subunits of PRC1.6 as interaction partners of RBPJ. The interaction of PRC1.6 with RBPJ promotes the binding of RBPJ to chromatin. Specifically, RBPJ uses not only PRC1.6-subunit L3MBTL2 but also SUMO as an additional interaction surface to bind to Notch target genes. Cofactors L3MBTL2 and E2F6 as chromatin regulators can contribute to target gene specificity in both low and high Notch activity states. Thus, SUMO attached to the PRC1.6 complex may regulate gene expression via recruitment of specific transcription factors (Fig. 6).

The known RBPJ interacting proteins KyoT2 or RITA use a φWφP motif to contact the BTD domain of RBPJ [33]. Importantly, L3MBTL2 interacts with a different region of RBPJ, the N-terminal domain, suggesting a different mode of interaction. Since malignant-brain-tumor (MBT) containing proteins were described to bind to methylated lysine residues, it is possible that lysine methylation of RBPJ also plays a role in the recruitment mechanism. Further biochemical and structural work will be needed to dissect this fairly strong, direct interaction between RBPJ and L3MBTL2.

The PRC1.6 complex subunits are SUMOylated and in particular L3MBTL2 is highly SUMOylated at steady state [19, 37]. Here we show that not only the L3MBTL2/RBPJ but also the SUMO/RBPJ interactions are important at Notch target genes. A limitation of our biochemical experiments is that we address the two interactions, L3MBTL2/RBPJ and SUMO/RBPJ, only separately. It would be better to demonstrate that in vitro SUMOylated L3MBTL2 interacts with RBPJ. For this purpose, it would be interesting to involve L3MBTL2 SUMO E3 ligase-PIAS1, which also regulates RBPJ-associated coactivator MAML1 [39]. Despite this, we speculate that PRC1.6 components and SUMO moieties cooperate and form a scaffold at selected genes, which is favorable for certain transcription factors such as RBPJ to interact. The SUMO-interaction motif as well as nearby SUMO moieties could be used to increase the dwelling time at such promoters and the transcription factor is given more time to find its enhancer elements and/or interact with additional other transcription factors.

E-boxes within Notch target gene regulatory elements have been described to play a role for Notch-dependent transcription [40, 41]. Interestingly, the MGA/MAX heterodimer binds E-boxes, while E2F6/DP-1/2 binds to E2F recognition sequences [18], which are likely used for the initial docking of the PRC1.6 complex at Notch target genes. Globally PRC1.6 recruitment has been investigated by Stielow et al. [18] and it has been stated that the PRC1.6 component MGA and E2F6 are crucial for the targeting mechanism. One interpretation is that occupancy of RBPJ at Notch target genes is enhanced by a concerted action of possibly several additional transcription factors, such as E2F6 and MGA (see also Fig. 6). Subsequently, the E2F6-/MGA-associated cofactors that are also posttranslationally modified by SUMOylation form the favorable environment for RBPJ binding.

Regarding the question what happens to gene expression upon depletion of L3MBTL2 or other PRC1.6 components, it is likely that this depends on the signaling state of the given cell type (Fig. 6). In constitutive active T-ALL cells (high Notch activity), knockdown of L3MBTL2 leads to downregulation of Notch target genes. In the repressed state changes in gene expression are expected to be mild, since there are also additional alternative corepressor complexes that are able to maintain repression. Such mild effects on transcriptional repression could also be explained in another manner. The interactions between SUMO and RBPJ in sum are more important than the interaction of one particular PRC1.6 component. The assumption is that this complex is SUMOylated at multiple subunits. Proteome-wide SUMOylation studies support this notion [36, 37]. Here, not only L3MBTL2 but also E2F6, MGA, PCGF6, RING1 and RYBP are found to be possible targets of SUMO.

The mode of action of Polycomb group proteins is to write and read certain histone marks. The classical dogma according to our current understanding indicates that PRC1 and PRC2 marks, H2AK119ub and H3K27me3, respectively, are important in the recruitment of each other. Genome-wide ChIP-Seq analysis in K562 cells revealed no H3K27me3 mark at L3MBTL2 binding sites [15]. It was previously observed that depletion of MGA, which abrogates the binding of L3MBTL2 and E2F6, does not lead to a global reduction of H3K27me3 or H2AK119ub [18]. However, H2AK119ub mark was described by Trojer and colleagues to be dependent on L3MBTL2 occupancy [15]. At regulatory elements of Notch target genes, this dependency is also not observed. In our study the non-canonical PRC1.6 complex first binds to Notch target gene promoter regions and this in turn enhances RBPJ binding. This supports a scenario that the chromatin-environment, in this case the occupancy of PRC1.6, make it more likely that RBPJ is able to dwell longer at these particular regulatory sites.

### Conclusion

We propose that the SUMOylated PRC1.6 subunits interact with RBPJ, and this provides a means for Notch target gene specificity by the PRC1.6 complex. Since RBPJ contains a SUMO-interaction motif, a network of SIM-containing and SUMO-modified proteins could stabilize such an interaction. Our work is in support for a direct functional role of SUMO and SUMO-interacting proteins to regulate chromatin and transcription.

## Materials and methods

### Cell culture and transfection

Generation of the RBPJ depleted HeLa cells was previously described [42]. HEK293, HEK293T, Hela and Hela-S cells were grown in Dulbecco’s Modified Eagle Medium (DMEM, Gibco 61965–059) supplemented with 10% fetal bovine serum (FBS, Pan Biotech) and penicillin/ streptomycin (Gibco). Mouse leukemia pre-T cells were grown in Iscove’s Modified Dulbecco Medium (IMDM, Gibco 21980–065) and supplemented with 2% of FBS (Pan Biotech), 0.3 mg/ml Primatone, nonessential amino acids (Gibco), 5 mg/l insulin (Merck) and penicillin/streptomycin (Gibco). All cell lines were grown at 37 °C under 5% CO_2_.

HEK293 and HEK293T cells were transiently transfected using linear polyethylenimine (PEI, Polysciences, 23966–1) or with the ProFection mammalian transfection system (Promega). For the polyethylenimine method cells (2.5 × 10^6^) were seeded on 10 cm plate in 10 ml of medium and incubated for 16-24 h. 14 μl of linear PEI was diluted in 309 µl of PBS, 20 µg of DNA were mixed with 325 µl of PBS, and combined together with diluted PEI. After 30 min of incubation in room temperature, DNA solution was added to the cells dropwise. The medium was changed to fresh one after 6 h of incubation in 37 °C.

For heat shock treatment cells were incubated for 1 h at 42 °C, followed by a recovery for 1 h at 37 °C.

For Luciferase assays, 20 × 10^4^ Hela cells were seeded in 48-well plates and transfected using Lipofectamine 2000 reagent (Thermo Fisher) according to manufacturer’s instructions.

### Lentiviral shRNA Knockdown in suspension cells

HEK293T cells (2.5 × 10^6^) were transfected using linear PEI as described above. 3.33 μg of desired plasmid DNA, 2,5 μg of psPAX and 1 μg of pMD were used. 48 h after transfection, supernatant was collected, filtered and 2 µg/ml of polybrene (Merck, H9268) was added. Supernatant containing viral particles was then used to infect Beko cells (5 × 10^5^) by centrifugation (1800 rpm, 45 min, 37 °C). Infection was repeated after 6, 24 and 30 h. Cells were selected with 1 µg/ml Puromycin (Serva, 33835) 24 h after the last infection. Knockdown experiments were performed using shRNA library (Merck).

### Generation of CRISPR/Cas9 mediated depletion cells

HEK293 cells were transfected using linear PEI either with empty vector px459 V2.0 pSpCas9(BB)-2A-Puro (WT) or with the same vector containing sgRNAs targeting the coding region of RBPJ (ENSE00003633263).

### Inhibitor treatment

HEK293 cells were treated with 10 µM SUMO inhibitor ML-792 (Medkoo, 407886) for 24 h. DMSO was used as a control.

### Antibodies

The following antibodies were used in this study: anti-L3MBTL2 (Active Motif, 39,569), anti-E2F6 (Diagenode, C15410314), anti-RBPJ (Cell signaling, 5313), rabbit IgG (Diagenode, C15410206), mouse IgG (Santa Cruz, sc2025), anti-Flag (Merck, F3165, F4042), anti-Flag HRP (Merck, A8592), anti-GFP (Merck/Roche, 11,814,460,001), anti-VP16 (Santa Cruz, sc-7545), anti-GAPDH (Abcam, ab8245), anti-TBP (Abcam, ab818), anti-GST (kind gift from Dr. M.L. Schmitz), anti-SUMO2/3 (Abcam, ab81371), anti-VINCULIN (Abcam, ab130007), anti-mouse IgG-HRP (Cell signaling, 7076 or Amersham NXA931), anti-rabbit IgG HRP (Cell signaling, 7074), anti-rat IgG HRP (Jackson ImmunoResearch, 112-035-072), sheep anti-mouse IgG HRP (GE Healthcare, NA931V), anti-Flag (M2) conjugated agarose beads (Merck, A2220), Hemaglutinin (HA) (Covance, MMS-101P).

### Protein extracts and western blotting

Whole cell extracts from HEK293 and HEK293T cells were obtained by washing cells in PBS and scraping them from the plate. The cells were washed twice in ice cold PBS and resuspended in extraction buffer (10 mM Tris/HCl [pH 7.5], 150 mM NaCl, 0.5 mM EDTA, 0.5% NP-40) freshly supplemented with 10 mM NaF, 0.5 mM sodium orthovanadate, 1 mM PMSF and 1 × protease inhibitor cocktail mix. After 30 min of incubation cells were centrifuged 20,000 rpm for 10 min in 4 °C. Protein concentrations were determined using Bradford assay (Merck). Equalled lysates were subjected for immunoprecipitation or boiled in SDS loading buffer and analysed by Western blotting.

Western blotting was performed by SDS-PAGE and proteins were transferred to nitrocellulose membranes using wet transfer. Membranes were blocked in 5% skimmed milk and subjected to overnight incubation with primary antibodies. After extensive washings in TBST (1 × TBS, 0.1% Tween 20), incubation with secondary antibody coupled to HRP was performed. The membranes were washed again using TBST and results were visualized using ECL solution and Vilber Fusion FX7 system.

### Co-immunoprecipitation

HEK293T cells were co-transfected with desired plasmids using linear PEI and harvested 48 h after the transfection. Protein levels after extraction (described above) were analysed using Bradford assay and equalized. GFP TRAP (Chromotek) beads were equilibrated according to the manufacture’s protocol. Protein extracts were than incubated with GFP TRAP beads for 1 h with rotation at 4 °C. Beads were centrifuged (2500×*g*, 5 min, 4 °C) and washed three times in Dilution Buffer (10 mM Tris/HCl [pH 7.5], 150 mM NaCl, 0.5 mM EDTA).After removing the supernatant, the beads were resuspended in SDS loading buffer and boiled.

### GST proteins purification and GST pulldown

GST fusion proteins were expressed in *Escherichia coli* strain BL21. After purification, the lysates from whole bacterial cells were stored at − 80 °C. Proteins were in vitro translated in presence of [^35^S] methionine using rabbit reticulocyte lysate system (Promega L4610). GST and GST fusion proteins were immobilized on Glutathione Sepharose beads and incubated with buffer A (40 mM HEPES [pH 7.5], 0.2 mM EDTA, 5 mM MgCl2, 100 mM KCl, 0.5% NP-40) with rotation for 1 h at 4 °C. Beads were washed one time with buffer A, two times with buffer B (40 mM HEPES [pH 7.5], 0.2 mM EDTA, 5 mM MgCl2, 300 mM KCl, 0.5% NP-40) and one time with PBS. Beads were boiled with SDS loading buffer and proteins were separated in SDS-PAGE. Dried gels were exposed to X-ray films (GE Healthcare).

### Modified GST pulldown

HEK293T cells were transfected using the desired expression plasmids. After 48 h the cells were collected and lysed in a lysis buffer. Protein extracts were analysed using Bradford assay and protein concentration was equalized among the samples. Glutathione Sepharose beads were washed three times (3000 rpm, 3 min, 4 °C) in ice cold PBS and then used to immobilize GST fusion proteins during 30 min incubation with rotation at 4 °C. After washing the beads 3 times, the protein extracts were added to the beads and incubated for 4 h with rotation at 4 °C. Beads were washed five times, 10 min with rotation at 4 °C, in ice cold PBS and boiled with SDS loading buffer.

### In vitro protein transcription/translation (TNT-assay)

In vitro protein translations were performed using the TNT-assay from Promega according to the manufacturer's instructions. Expression of translated RBPJ proteins (wt, IV/AA, R218H) was monitored by western blotting.

### Electro mobility shift assay (EMSA)

Reticulocyte lysates (2 μl and 4 μl) from in vitro translations (see above) were used for the EMSAs. Binding reaction was performed in a buffer consisting of 10 mM Tris–HCl (pH 7.5), 100 mM NaCl, 0.1 mM EDTA, 0.5 mM DTT and 4% glycerol. For binding reaction, 10 ng (0.02 U) poly(dI-dC) (GE Healthcare) and approximately 0.5 ng of ^32^P-labelled oligonucleotides were added. The sequence of the double-stranded oligonucleotide FO-233F/FO-233R (Additional file 3: Table S2) corresponds to the two RBPJ-binding sites within the EBV TP-1 promoter. Where indicated an anti-Flag antibody (M5, Merck) where added to the binding reaction to analyse specificity of binding (supershift). DNA–protein complexes were separated in SDS-PAGE. Gels were dried and exposed to X-ray films (GE Healthcare).

### Luciferase assays

For reporter gene assays Hela cells were transfected using Lipofectamine (as described above) with 250 ng of the reporter construct pGa981/6 (12 × CSL-RE-Luc) alone or together with 10 ng of expression plasmid. After 24 h luciferase activity was determined from at least six independent experiments from 20 μl of cleared lysate. Measurements were performed using a LB 9501 luminometer (Berthold) and the luciferase assay system from Promega.

### Fluorescence microscopy

HeLa cells were plated (1 × 10^5^ cells/cm^2^) on chamber coverslips (Nunc). After 18 h, cells were transfected with 150 ng of expression plasmids for the specific GFP-fusion proteins. After 24 h, cells were rinsed with PBS, fixed with 4% paraformaldehyde and permeabilized with 0.1% Triton X-100. Specimens were embedded in ProLong© Gold antifade reagent (Invitrogen) supplemented with [2-(4-carbamimidoylphenyl)-1H-indol-6-carboximidamide] (DAPI) and stored at 4 °C overnight. Cells were imaged using a fluorescence microscope (IX71, Olympus) equipped with a digital camera (C4742, Hamamatsu) and a 100-W mercury lamp (HBO 103 W/2, Osram) using the following filter sets: GFP detection, ex: HQ470/40, em: HQ525/50, DAPI detection, ex: HQ360/40, em: HQ457/50.

### Oligonucleotides and constructs

The complete list of oligonucleotides used in this study is shown in Additional file 3: Table S2.

For complex purification of RBPJ, human RBPJ was cloned into pENTR/D-Topo via PCR and then transferred into a lentiviral destination vector containing an N-terminal Flag-HA-tag using Clonase (Gateway system).

The GST-L3MBTL2 expression plasmid was generated from pCMV tag4A L3MBTL2 plasmid (kindly provided by Dr. G. Suske). Fragment digested using BamHI and XhoI restriction sites was inserted into pGEX6P1 plasmid (GE Healthcare) pre-digested with the same restriction sites.

pGEX-4T-1 GST, GST SUMO1, GST SUMO2, GST SUMO3 fusion proteins were kindly provided by Dr. M.L. Schmitz.

GST-RBPJ fusion protein was previously described [35].

pcDNA 3.1 Flag hL3MBTL2 ΔN (aa179-705), C (aa604-705), N (aa1-178), ΔC (aa1-603) fragments used for GST pull down experiments were generated by PCR-amplification, primers used for PCR are listed in Additional file 3: Table S2. PCR products were first cloned into pSC-A-amp/kan (Agilent Technologies, 2402055), digested with desired restriction enzymes and inserted into pre-digested pcDNA 3.1 Flag2 (Invitrogen).

pN3-3xFLAG-C-Terminus L3MBTL2 was kindly provided by Dr. G. Suske. pcDNA hRBPJ N/B (aa1-315), B (aa166-334), B/C (aa166-487) fragments and pcDNA hRBPJ NTD (aa1-165) were generated by PCR as previously described [30, 43].

mRBPJ ∆NTD (aa206-526 which corresponds to human fragment aa166-487) was generated by PCR amplification, primers used for PCR are listed in Additional file 3: Table S2. PCR products were first cloned into pSC-A-amp/kan (Agilent Technologies, 2402055), digested with desired restriction enzymes and inserted into pre-digested pcDNA EGFP mRBPJ WT.

To generate expression vectors for the RBPJ IV/AA mutant, specific mouse RBPJ fragments of 721 bp EcoRV/KpnI were synthetized at BioCat GmbH and inserted using corresponding restriction sites into predigested pcDNA3 mRBPJ EGFP (wt) and pcDNA3-Flag-mRBPJ(wt). All plasmids were analysed by sequencing.

### RNA extraction, reverse transcription and qPCR

Total RNA was isolated using Trizol reagent (Ambion, 15596018), following the manufacturer’s instructions. 1 µg of RNA was used for generating cDNA using random hexamers and reverse transcriptase M-MuLV (NEB). qPCR analysis were performed using cDNA, Absolute QPCR ROX Mix (Thermo Scientific, AB-1139), gene-specific oligonucleotides, double dye probes (see Additional file 3: Table S2). StepOnePlus (Applied Biosystems) was used as a sequence detector system. Data were normalized to a housekeeping gene.

### ChIP-qPCR

The cells were collected in ice cold PBS and crosslinked at room temperature for 1 h in 10 mM DMA. After one washing in PBS the cells were additionally crosslinked for 30 min in 1% formaldehyde. Crosslinking was stopped by adding 1 M Glycine (pH 7.5) for 5 min. After two washings in PBS, cells were pelleted at 3000 rpm, 3 min in 4 °C and lysed in 1 ml of SDS Lysis Buffer for 10 min on ice. The lysates were sonicated in Covaris System (28 cycles) and the chromatin was sheared to fragments ranging from 200 to 1000 bp. After measuring concentration, sheared chromatin was aliquoted and stored for further experiments at − 80 °C.

25 μg to 50 μg of chromatin were diluted in ratio 1:6 in ChIP dilution buffer (0.01% SDS, 1.2 mM EDTA, 16.7 mM Tris HCl [pH 8.1], 167 mM NaCl, 1,1% Trition X-100) and precleared with pre-saturated protein-A-Sepharose beads (GE Healthcare 17-5280-02) for 30 min at 4 °C with rotation. Protein-A-Sepharose beads were pelleted at 3000 rpm, 3 min in 4 °C. Diluted chromatin was transferred to a new tube. The input was transferred to a separate tube and stored in 4 °C for the next day. Desired antibodies used for immunoprecipitation were added to diluted chromatin and incubated overnight in 4 °C with rotation. Antibody–protein–DNA complexes were bound to saturated protein A beads and washed each time for 5 min at 4 °C with rotation. The washings steps were as follows: 1 × low salt washing buffer (20 mM Tris/HCl [pH 8.1], 150 mM NaCl, 2 mM EDTA, 0.1% SDS, 1% Triton X-100), 1 × high salt washing buffer (20 mM Tris/HCl [pH 8.1], 500 mM NaCl, 2 mM EDTA, 0.1% SDS, 1% Triton X-100), 1 × LiCl washing buffer (10 mM Tris/HCl [pH 8.1], 250 mM LiCl, 1 mM EDTA, 1% NP-40), 3 × TE buffer (10 mM Tris/HCl [pH 7.9], 1 mM EDTA) or 1 × low salt washing buffer, 2 × high salt washing buffer, 2 × LiCl washing buffer, 3 × TE buffer in case of RBPJ ChIP. DNA bound to the proteins was eluted in two steps using freshly prepared elution buffer (1% SDS, 0.1 M NaHCO3) for 15 min at room temperature. After combining two elutions, 20 μl of 5 M NaCl was added and samples were reverse crosslinked overnight at 65 °C. Bound proteins were digested using Proteinase K for 1 h at 45 °C. DNA was purified using phenol–chloroform extraction and precipitated overnight at − 20 °C in isopropanol in presence of tRNA and glycogen. Samples were centrifuged 13,000 rpm, 30 min at 4 °C and washed in 70% ethanol. After centrifugation samples were dried in the concentrator and resuspended in 20 μl of TE buffer.

### RBPJ complex purification and mass spectrometry

Flag-HA-tagged human RBPJ was expressed after lentiviral infection of HeLa-S. The cells were expanded to 8L and harvested. All following steps were performed at 4 °C. The cells were lysed via incubating with 5 cell pellet volume hypotonic buffer (10 mM Tris/HCl, pH 7.3, 10 mM KCl, 1.5 mM MgCl2, 0.2 mM PMSF and 10 mM β-Mercaptethanol, Protease inhibitor) for 30 min and douncing. The mixture was centrifuged (4000 rpm) and the nuclei pellet was washed twice with hypotonic buffer. Nuclear extract was made by low salt/high salt extraction. The nuclei were resuspended in 1 nuclei pellet volume low salt buffer (20 mM Tris/HCl, pH7.3, 20 mM KCl, 1.5 mM MgCl2, 0.2 mM EDTA, 25% glycerol, 10 mM β-mercaptoethanol, protease inhibitor). Subsequently, 0.6 nuclei pellet volume high salt buffer (20 mM Tris/HCl, pH 7.3, 1.2 M KCl, 1.5 mM MgCl2, 0.2 mM EDTA, 25% Gyclerol, 10 mM β-Mercaptethanol, Protease inhibitor) was added dropwise under stirring. After adding the high salt buffer, the mixture was incubated for further 30 min, stirring. The mixture was centrifuged at 13,000 rpm, and the supernatant was taken. The gained nuclear extract was split into two fractions. To one fraction the wild type (2 µg) and mutated oligonucleotide (16 µg) was added. The other fraction remained untouched (see Fig. 1a, c). Both fractions were dialyzed overnight in (20 mM Tris/HCl, pH7.3, 100 mM KCl, 0.2 mM EDTA, 20% Gyclerol, 10 mM β-Mercaptethanol, 1 mM DTT, Protease inhibitor). The RBPJ complexes were then purified using anti-Flag (M2) conjugated agarose beads by incubation in TAP buffer (50 mM Tris–HCl pH 7.9, 100 mM KCl, 5 mM MgCl2, 10% glycerol, 0.1% NP-40, 1 mM DTT, and protease inhibitors) for 4 h and 3 times washing with TAP buffer. Flag-HA-RBPJ was eluted from the beads with Flag peptides. The obtained samples were TCA precipitated and peptides were identified via LC–MS/MS at the Taplin Core facility (Harvard Medical School).

## Supplementary Information

**Additional file 1: Table S1.** The complete list of RBPJ-associated proteins identified by mass spectrometry using an oligonucleotide-assisted complex purification approach.

**Additional file 2: Figures S1.** (a) HEK293T cells were transiently transfected with GFP-RBPJ plasmid. Cell lysates were subjected to GFP immunoprecipitation. Control cells were transfected with pcDNA GFP plasmid. (b) GST-RBPJ fusion protein was expressed in bacteria and purified. Fragments of L3MBTL2 were labeled with [^35^S] methionine, in vitro translated in RRL system and incubated with GST-RBPJ fusion protein immobilized on sepharose beads. (c) HEK293T cells were co-transfected with Flag-L3MBTL2 C-term and GFP-RBPJ. Protein extracts of cell lysates were subjected to GFP immunoprecipitation. Control cells were transfected with pcDNA GFP plasmid. (d) GST-L3MBTL2 fusion protein was expressed in bacteria and purified. Fragments of RBPJ were radioactively labeled with [^35^S] methionine, in vitro translated in RRL system and incubated with GST-L3MBTL2 fusion protein immobilized on sepharose beads. (e) GST-L3MBTL2 fusion protein was expressed in bacteria and purified. RBPJ NTD fragment was labeled with [^35^S] methionine, in vitro translated in RRL system and incubated with GST-L3MBTL2 fusion protein immobilized on sepharose beads. (f) Protein extracts of Beko cell lysates after extraction were subjected to immunoprecipitation with either L3MBTL2 antibody or IgG as a control. Immunoprecipitates were analysed by Western blotting with anti-L3MBTL2 and anti-RBPJ antibody. **Figure S2.** (a) Chromatin Immunoprecipitation of endogenous RBPJ and its binding at regulatory elements of Notch target genes in wild type and in RBPJ depleted cells (clone A12). G*ene Desert* served as a negative control (CTRL). The mean of at least three independent biological replicates ± SD. (b) Western Blot analysis of endogenous L3MBTL2 in wild type HEK293 and in L3MBTL2-depleted cells. TBP served as a loading control. (c) Western Blot analysis of endogenous E2F6 in wild type HEK293 and in E2F6-depleted cells. VINCULIN served as a loading control. (d) Schematic representation of the targeting strategy for generating CRISPR/Cas9 mediated RBPJ depletion in HEK293 cells (Exon: ENSE00003633263). (e) Western Blot analysis of endogenous RBPJ in wild type HEK293 and in RBPJ-depleted cells (clones A2 and A12). *GAPDH* served as a loading control. (f) mRNA level of *RBPJ* in CRISPR/Cas9 mediated RBPJ depletion in HEK293 (clone A12). Data was normalised to *TBP*, *GAPDH* served as a positive control. (*P < 0.05, **P < 0.01, ***P < 0.001, [NS] not significant, unpaired Student's t-test). The mean of at least three independent biological replicates ± SD is shown. **Figure S3.** (a) ChIP qPCR analysis of SUMO2/3 enrichment at regulatory elements of Notch target genes in HEK293 cells. (b) GST-SUMO2 fusion protein was expressed in bacteria and purified. HEK293T cells were transiently transfected with GFP-RBPJ wild type or GFP-RBPJ ∆NTD mutant and whole cell extracts were incubated with GST fusion protein immobilized on sepharose beads. (c, upper) Subcellular localisation of GFP-RBPJ wt and GFP-RBPJ IV/AA mutant. Hela cells were transiently transfected with GFP-RBPJ wt or GFP-RBPJ IV/AA mutant and fixed 24 h after transfection. (c, lower) Western blot show slightly reduced expression of the GFP-RBPJ (IV/AA) mutant. HeLa cells were transiently transfected with GFP-RBPJ expression vectors. 24 h after transfection cells, where lysed and expression of the GFP-fusions were analysed by western blotting. Actin expression served as a loading control. (d, upper) Transactivation capacities of RBPJ (wt) and RBPJ (IV/AA) mutant together with NICD. Hela^RBPJ−KO^ cells were cotransfected with NICD together with either Flag-RBPJ-wt or Flag-RBPJ IV/AA mutant and the 12 × CSL-RE-Luc reporter construct containing 12 RBPJ DNA binding sites upstream of the luciferase gene. The mean of at least four independent biological replicates ± SD is shown (ns, *p* ≥ 0.05; ***, *p* < 0.0001, unpaired students T-test). (d, lower) Western blot show slightly reduced expression of the Flag-RBPJ (IV/AA) mutant. HeLa cells were transiently transfected with Flag-RBPJ expression vectors. 24 h after transfection cells where lysed and expression of Flag-tagged proteins were analysed by western blotting. Actin expression served as a loading control. **Figure S4.** (a) Schematic representation of the SUMO pathway. SUMO after establishing ATP dependent thioester bond with heterodimeric Aos1/Uba2 SUMO activating E1 enzyme (SAE), is transferred to E2 enzyme (Ubc9) and subsequently bound to the substrate by E3 SUMO ligase. ML-792 selectively blocks E1 (SAE). (b) HEK293T cells were co-transfected with Flag-L3MBTL2 and GFP-RBPJ. The cells were treated for 24 h with 10 µM ML-792 or the vehicle. Cell lysates after extraction were subjected for GFP immunoprecipitation. Control cells were transfected with pcDNA GFP plasmid. (c) HEK293T cells were co-transfected with Flag-RBPJ and GFP-NICD1. The cells were treated for 24 h with 10 µM ML-792 or DMSO. Cell lysates after extraction were subjected for GFP immunoprecipitation. Control cells were transfected with pcDNA GFP plasmid. (d) HEK293T cells were co-transfected with Flag-L3MBTL2 and GFP-RBPJ. Heat shocked cells were incubated for 1 h at 42 °C and allowed to recover for 1 h at 37 °C. Cell lysates after extraction were subjected to GFP immunoprecipitation. Control cells were transfected with pcDNA GFP plasmid.

**Additional file 3: Table S2.** Oligonucleotides and primers used for the study.

## Data Availability

All data generated or analysed during this study are included in this published article and its Additional files.

## Abbreviations

BTD: Beta trefoil domain
CBF1: C promoter binding factor
ChIP: Chromatin immunoprecipitation
CSL: *Homo sapiens* CBF1, *Drosophila melanogaster* Suppressor of Hairless, and *Caenorhabditis elegans* Lag-1
CTD: C-terminal domain
CTRL: Control
E2F6: E2F Transcription Factor 6
EMSA: Electro Mobility Shift Assay
EV: Empty vector
EZH2: Enhancer of Zeste 2
GFP: Green fluorescent protein
GST: Glutathione S transferase
HDAC: Histone deacetylase
HES: Hairy/enhancer of split
KDM1A/LSD1: Lysine demethylase 1A
L3MBTL2: Lethal (3) malignant brain tumor-like protein 2
L3MBTL3: Lethal (3) malignant brain tumor-like protein 3
MAML: Mastermind-like
MBT: Malignant brain tumor
MS: Mass spectrometry
NCoR: Nuclear receptor corepressor
NRARP: Notch-regulated ankyrin-repeat protein
NTD: N-terminal domain
MGA: MAX gene associated
PCGF6: Polycomb Group Ring Finger 6
PD: Pulldown
PRC: Polycomb repressive complex
PTMs: Posttranslational modifications
RITA: RBPJ Interacting and tubulin associated
RRL: Rabbit reticulocyte lysate
SAE: SUMO1 Activating Enzyme Subunit
SCR: Scrambled
SD: Standard deviation
SHARP: SMRT and HDAC associated repressor protein
shRNA: Short hairpin RNA
SIM: SUMO interacting motif
SUMO: Small ubiquitin-like modifier
T-ALL: T-cell acute lymphoblastic leukemia
TBP: TATA-binding protein
UACA: Uveal autoantigen with coiled-coil domains and ankyrin repeats
UBA2: Ubiquitin-like modifier activating enzyme 2
UBC9: Ubiquitin conjugating enzyme 9
WDR5: WD repeat domain 5
WT: Wild type
