# Supplementary figures and images for: SUMOylated non-canonical polycomb PRC1.6 complex as a prerequisite for recruitment of transcription factor RBPJ
Source: Epigenetics Chromatin. 2021 Jul 31;14:38. doi: 10.1186/s13072-021-00412-9 (PMC8325870; doi:10.1186/s13072-021-00412-9)

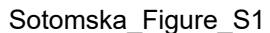

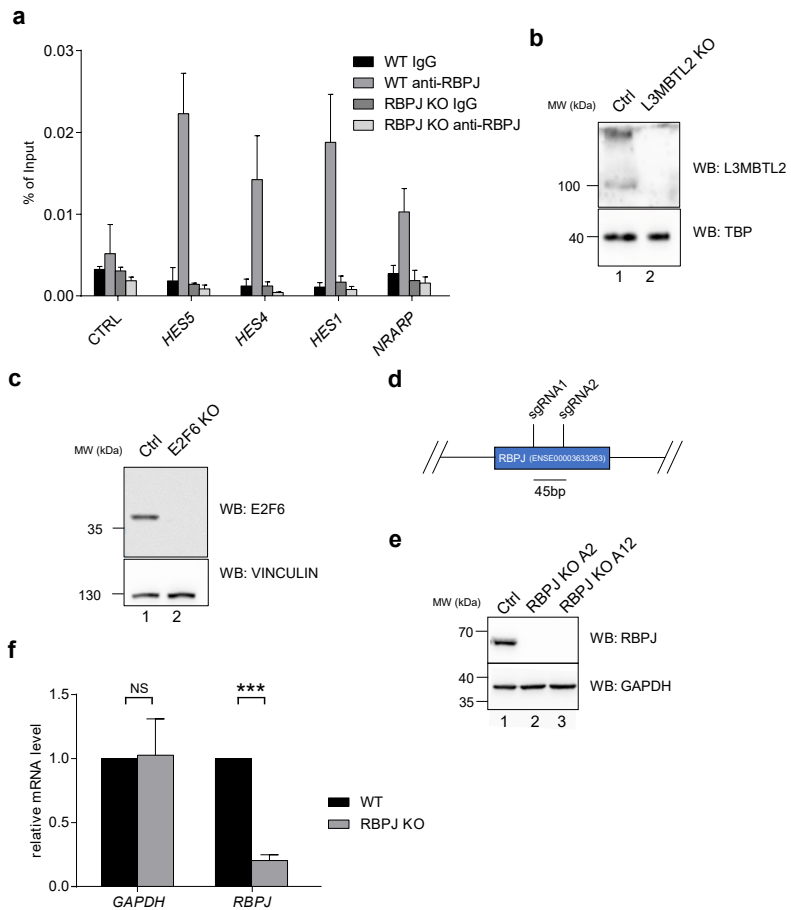

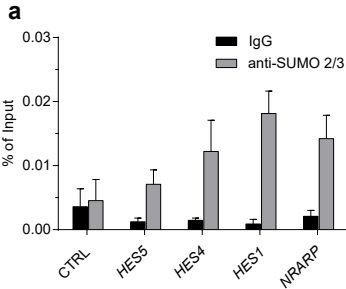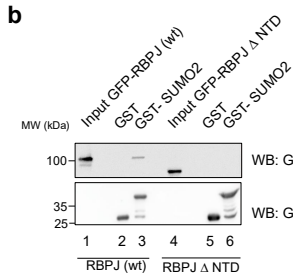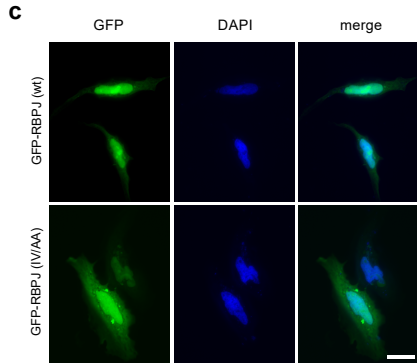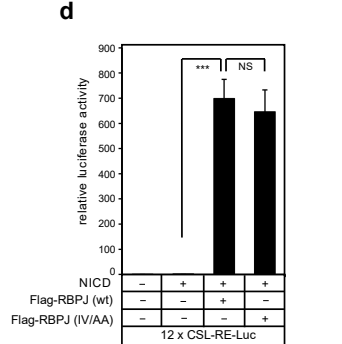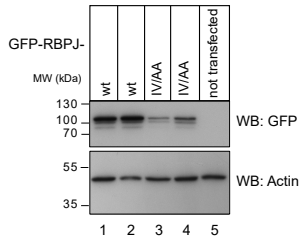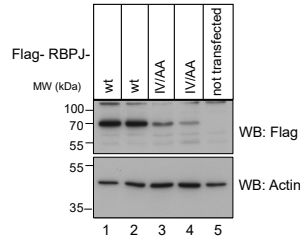

**a**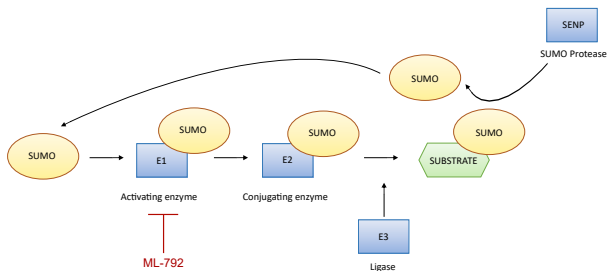**b**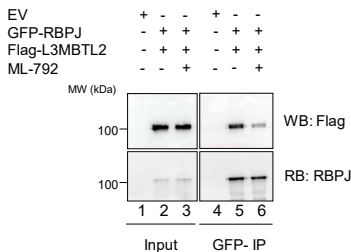**c**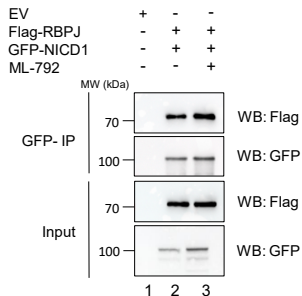**d**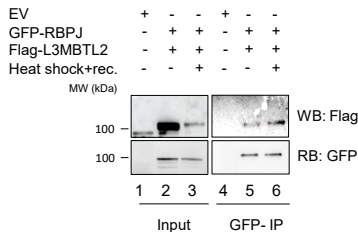

Supplement: Supplementary file 2 — Additional file 2: Figures S1. (a) HEK293T cells were transiently transfected with GFP-RBPJ plasmid. Cell lysates were subjected to GFP immunoprecipitation. Control cells were transfected with pcDNA GFP plasmid. (b) GST-RBPJ fusion protein was expressed in bacteria and purified. Fragments of L3MBTL2 were labeled with [35S] methionine, in vitro translated in RRL system and incubated with GST-RBPJ fusion protein immobilized on sepharose beads. (c) HEK293T cells were co-transfected with Flag-L3MBTL2 C-term and GFP-RBPJ. Protein extracts of cell lysates were subjected to GFP immunoprecipitation. Control cells were transfected with pcDNA GFP plasmid. (d) GST-L3MBTL2 fusion protein was expressed in bacteria and purified. Fragments of RBPJ were radioactively labeled with [35S] methionine, in vitro translated in RRL system and incubated with GST-L3MBTL2 fusion protein immobilized on sepharose beads. (e) GST-L3MBTL2 fusion protein was expressed in bacteria and purified. RBPJ NTD fragment was labeled with [35S] methionine, in vitro translated in RRL system and incubated with GST-L3MBTL2 fusion protein immobilized on sepharose beads. (f) Protein extracts of Beko cell lysates after extraction were subjected to immunoprecipitation with either L3MBTL2 antibody or IgG as a control. Immunoprecipitates were analysed by Western blotting with anti-L3MBTL2 and anti-RBPJ antibody. Figure S2. (a) Chromatin Immunoprecipitation of endogenous RBPJ and its binding at regulatory elements of Notch target genes in wild type and in RBPJ depleted cells (clone A12). Gene Desert served as a negative control (CTRL). The mean of at least three independent biological replicates ± SD. (b) Western Blot analysis of endogenous L3MBTL2 in wild type HEK293 and in L3MBTL2-depleted cells. TBP served as a loading control. (c) Western Blot analysis of endogenous E2F6 in wild type HEK293 and in E2F6-depleted cells. VINCULIN served as a loading control. (d) Schematic representation of the targeting strategy for g [file 13072_2021_412_MOESM2_ESM.pdf]
